# Patterns of multimorbidity in primary care electronic health records: A systematic review

**DOI:** 10.1177/26335565231223350

**Published:** 2024-01-30

**Authors:** Giorgi Beridze, Ahmad Abbadi, Joan Ars, Francesca Remelli, Davide L Vetrano, Caterina Trevisan, Laura-Mónica Pérez, Juan A López-Rodríguez, Amaia Calderón-Larrañaga

**Affiliations:** 1 Department of Neurobiology, Care Sciences and Society, Karolinska Institutet and Stockholm University, 193201 Aging Research Center, Stockholm, Sweden; 2 RE-FiT Barcelona Research group, Vall d'Hebron Institute of Research (VHIR) and Parc Sanitari Pere Virgili, Barcelona, Spain; 3 Medicine Department, Universitat Autònoma de Barcelona, Barcelona, Spain; 4 Department of Medical Sciences, 9299 University of Ferrara, Ferrara, Italy; 5 Stockholm Gerontology Research Center, Stockholm, Sweden; 6 Research Unit, Primary Health Care Management, Madrid, Spain; 7 Department of Medical Specialties and Public Health, Faculty of Health Sciences Rey Juan Carlos University, Madrid, Spain; 8 Research Network on Chronicity, Primary Care and Health Promotion (RICAPPS), Carlos III Health Institute, Madrid, Spain

**Keywords:** Multimorbidity, electronic health records, primary care

## Abstract

**Background:**

Multimorbidity, the coexistence of multiple chronic conditions in an individual, is a complex phenomenon that is highly prevalent in primary care settings, particularly in older individuals. This systematic review summarises the current evidence on multimorbidity patterns identified in primary care electronic health record (EHR) data.

**Methods:**

Three databases were searched from inception to April 2022 to identify studies that derived original multimorbidity patterns from primary care EHR data. The quality of the included studies was assessed using a modified version of the Newcastle-Ottawa Quality Assessment Scale.

**Results:**

Sixteen studies were included in this systematic review, none of which was of low quality. Most studies were conducted in Spain, and only one study was conducted outside of Europe. The prevalence of multimorbidity (i.e. two or more conditions) ranged from 14.0% to 93.9%. The most common stratification variable in disease clustering models was sex, followed by age and calendar year. Despite significant heterogeneity in clustering methods and disease classification tools, consistent patterns of multimorbidity emerged. Mental health and cardiovascular patterns were identified in all studies, often in combination with diseases of other organ systems (e.g. neurological, endocrine).

**Discussion:**

These findings emphasise the frequent coexistence of physical and mental health conditions in primary care, and provide useful information for the development of targeted preventive and management strategies. Future research should explore mechanisms underlying multimorbidity patterns, prioritise methodological harmonisation to facilitate the comparability of findings, and promote the use of EHR data globally to enhance our understanding of multimorbidity in more diverse populations.

## Introduction

Owing to increases in life expectancy and improvements in medical care, more people than ever are living with multimorbidity, which is commonly defined as the coexistence of multiple chronic conditions in an individual.^1^ Depending on the population studied and the list of conditions assessed, prevalence estimates may be as high as 41% in the general population^2^ and 89% in older adults aged 60 years and above.^3^ Individuals with multimorbidity often require complex management plans and are at a higher risk of adverse health outcomes, including hospitalisation,^4^ functional impairment,^5^ and poor quality of life.^6^

While multimorbidity has traditionally been operationalised as counts or weighted indices of chronic conditions, the importance of considering the non-random clustering of conditions has been increasingly recognised.^7^ It has been claimed that the study of multimorbidity patterns has the potential to facilitate a shift from a single-disease paradigm to a more holistic and patient-centred approach to care.^8,9^ Several statistical methods have been employed to identify clusters of conditions that co-occur more frequently than chance, providing insight into possible underlying mechanisms and highlighting potential avenues for intervention.^10^ Such patterns have also been shown to have strong discriminative capacities for a wide spectrum of important health-related outcomes, including healthcare utilisation,^11^ institutionalisation,^12^ and the risk of developing health conditions that can profoundly impact individuals' quality of life (e.g. dementia,^13^ frailty^14^ and disability^15^).

The generalisability of patterns across various settings and populations can be challenging due to the substantial variations in data sources and the operationalisation of chronic conditions.^16^ Indeed, hospital data may not capture the full burden of multimorbidity, as chronic conditions that are less acute or unrelated to the cause of hospitalisation may be underreported. On the other hand, community-based studies may focus on a priori shorter lists of conditions, fail to capture individuals with a higher burden or more complex combinations of conditions, or have less reliable data if self-reported data cannot be cross-checked with clinical data.^17^

The increased availability of data from electronic health records (EHR) over the last decade has provided researchers access to large-scale primary care data, potentially paving the way for a more comprehensive understanding of multimorbidity patterns in the general population. Indeed, given the longitudinal and generalist nature of the care provided by primary care physicians, such data sources would likely capture a broader spectrum of health conditions.^18^ Furthermore, primary care data may increase the internal and external validity of the findings by including larger and more diverse populations, thus enabling real-world descriptions of multimorbidity patterns.^19^

Two reviews have previously attempted to systematically summarise the literature on multimorbidity patterns. Prados-Torres et al.^20^ used a qualitative approach to identify replicable patterns, while Busija et al.^21^ used a quantitative approach (multidimensional scaling). Both studies identified two multimorbidity patterns (cardiovascular and metabolic diseases, and mental health conditions) that coexisted across studies using different populations, data sources, and clustering methods. The overall conclusion of both reviews, however, was that there was significant heterogeneity in the methodological criteria applied to study multimorbidity patterns, particularly in the selection of chronic conditions included in the studies. To address the latter limitation and update the current state of knowledge on multimorbidity patterns, we aimed to conduct a systematic review of multimorbidity patterns, with a specific focus on studies based on primary care EHR data.

## Methods

The reporting of this systematic review is based on the Preferred Reporting Items for Systematic Reviews and Meta-Analyses (PRISMA) 2020 statement (Supplementary Table 1).

### Search strategy

A literature search was performed in the following databases: Medline (Ovid), Web of Science Core Collection (Clarivate), and CINAHL (EBSCO). Two librarians from Karolinska Institutet conducted the search after consultation with the authors on the most relevant terms and concepts. One librarian was responsible for developing the search and received feedback on terminology, operators, syntax, spelling, etc. from another librarian. The search was then translated into other databases, in part using Polyglot Search Translator.^22^ The strategies were proof-read by another librarian prior to execution. De-duplication was done using the method described by Bramer et al.^23^ One final, extra step was added to compare DOIs. The search combined Medical Subject Headings (MeSH) terms and free-text expressions related to multimorbidity and comorbidity (e.g. [Comorbidity]), patterns of diseases (e.g. [pattern*], [cluster*]), primary healthcare (e.g. [Primary Health Care], [family practice]), and electronic medical records (e.g. [electronic health records]). Databases were searched from their inception to April 26, 2022, and articles were restricted to English language based on the authors’ language competencies. The detailed search strategy is presented in Supplementary Tables 2A-2C.

### Inclusion criteria

The following criteria were used to screen the articles and include them in this study:

a. Original peer-reviewed research papers written in English.
b. Focus on identifying patterns of associative multimorbidity (i.e. non-random co-occurrence of health conditions).
c. Explicit description of the method(s) used for exploring multimorbidity patterns.
d. Focus on primary care populations and usage of EHRs as the data source.

### Exclusion criteria

Articles were excluded if any of the following criteria were met:

a. Descriptive measures of multimorbidity were the only focus of the study (e.g. studies based on the prevalence or count of health conditions).
b. One-to-one disease associations were the sole focus of the study, without identifying communities of co-occurring health conditions (e.g. studies based on disease combinations or network analysis).
c. The study focused only on grouping patients without reporting data on the co-occurrence of health conditions.
d. The study began with a selection of an index condition (i.e. all included participants had an index condition).
e. The study initially selected fewer than 10 conditions for analysis.
f. The study did not derive original patterns (i.e. used *a priori* defined patterns or patterns derived in another study).

### Selection of articles

After deduplication, references were imported into Covidence, a web-based collaboration software platform that streamlines the production of systematic and other literature reviews.^24^ Two authors (FR and JA) independently screened the titles and abstracts of the studies for eligibility based on pre-specified inclusion and exclusion criteria. Any discrepancies were resolved through a discussion with a third author (ACL).

Following title and abstract screening, a full-text review was conducted on all potentially eligible articles by a team of four authors (AA, FR, GB, and JA) working in duplicate. In cases where a study did not meet the inclusion criteria or met one or more exclusion criteria, it was excluded based on the first criterion that appeared in the inclusion or exclusion criteria. Additional reasons for exclusion were not specified. Any conflicts that arose during the full-text review were resolved by a third reviewer (AA, ACL, or GB), who was not part of that specific duplicate review pair.

### Data extraction

For articles that met all inclusion criteria and none of the exclusion criteria, information about study design and characteristics (e.g. author, year, title, country, aim, design, age, sex, and disease classification) and results (e.g. number of diseases, type of analysis, and patterns generated) were extracted. The conclusions of the authors were also reviewed. Data extraction was performed in duplicate by AA and JA, and conflicts were resolved by consensus or by a third author (GB).

### Quality assessment

A modified version of the Newcastle-Ottawa Quality Assessment Scale was used to evaluate the quality of the articles included in the study.^25^ Modifications were tailored to the specifics of our research question (e.g. EHR as a data source) and the types of studies included in the review. We removed the outcome evaluation item (assessment of the outcome) because there were no outcomes to be studied in our research question. The modified tool includes six criteria and allows for a score ranging from zero (minimum) to eight (maximum) stars. Subsequently, the studies were classified into three categories: poor (0-2 stars), moderate (3-5 stars), and high (6-8 stars). The modified quality assessment tool is available in the Supplementary Box.

The quality assessment was performed in duplicate by AA and JA. Disagreements were first addressed through a consensus meeting between the two authors. Any remaining disagreements were resolved by a third author (GB or ACL).

### Data synthesis

Owing to the high variability in the types of methods and sources of data, a narrative synthesis was performed. The extracted data were organised into tables, and the characteristics, methods, and resulting patterns of each study were evaluated. In cases where patterns were not explicitly named in the original studies, the review authors assigned names to each pattern based on the diseases that characterised them (whenever there were more than three overexpressed diseases in the pattern). Methodological approaches were compared, and the number and content of patterns were evaluated based on the type of analysis used. Additionally, the patterns generated from studies which stratified by age, sex, country of origin, or other variables were compared to those generated from studies that only presented overall patterns.

## Results

### Articles included in the review

**Figure 1.**
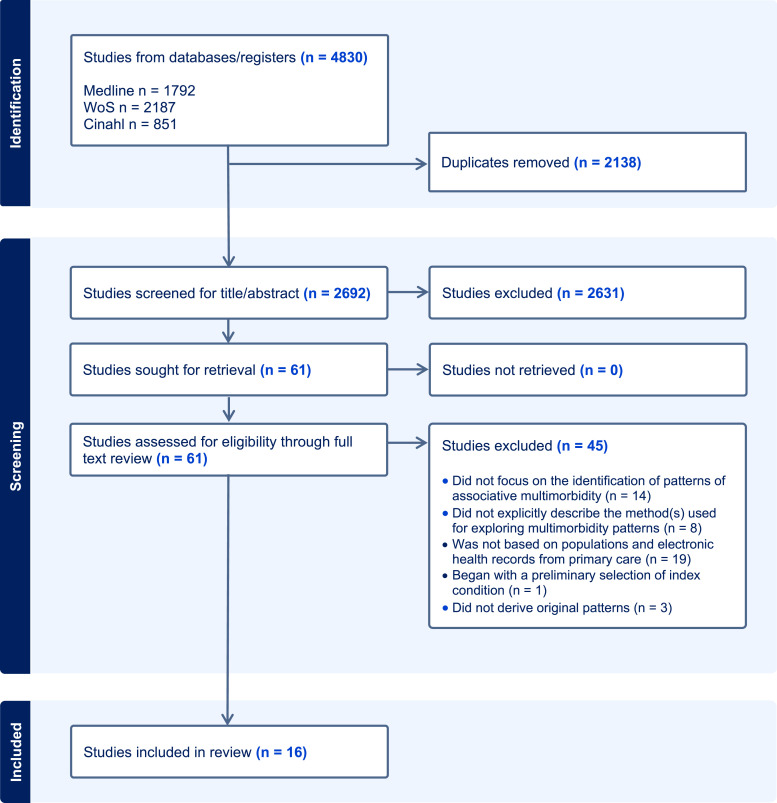
Flowchart of the study selection.

A total of 4,830 articles were initially identified through our search strategy, which were subsequently deduplicated, leaving 2,692 articles for further screening (Figure 1). Following title and abstract screening, 2,631 articles were excluded based on the predetermined eligibility criteria. Of the remaining 61 articles that underwent full-text screening, 16 met the eligibility criteria and were included in this review.^26–41^ The excluded studies did not identify multimorbidity patterns (n = 14), did not explicitly describe the methods used to generate multimorbidity patterns (n = 8), did not include populations or EHR data from primary care (n = 19), began with a preliminary selection of an index disease (HIV; n = 1), or did not derive original patterns (n = 3). The list of studies excluded during the full-text review, along with the reason for exclusion, is found in Supplementary Table 3.

### Quality assessment of included studies

The results of the quality assessment of the 16 studies included in this review are presented in Supplementary Table 4. The scores ranged from four to eight stars, with a median and mode of seven. Among the 16 studies, only one study received the lowest observed score of four stars, while two studies received five stars each. Only one study achieved the maximum score of eight stars. The remaining studies (12 of 16) scored either six or seven stars. The quality criterion that was most commonly lacking was “comparability” (i.e. stratification and/or adjustment for sociodemographic and other relevant factors). Overall, all studies had either moderate or high quality.

### Characteristics of included studies

Table 1 provides an overview of the characteristics of the 16 studies included in this systematic review. Most of the studies were conducted in Spain (n = 10), followed by two studies in the UK,^26,41^ and one each in Norway,^27^ Sweden,^29^ the Netherlands,^34^ and Mexico.^33^ Data availability ranged between the years 2005-2020. The number of primary healthcare centres included in the studies ranged from 4 to over 200, although some studies reported the number of general practitioners instead. The number of participants included across studies ranged from 813 to 3,740,528, with only one study having fewer than 1,000 participants^32^ and the remaining studies having more than 38,000 participants. Participants’ ages varied across studies, with some including all age groups and others restricting the participants to specific age groups (e.g. 45-64). The prevalence of multimorbidity (i.e. the coexistence of ≥2 chronic conditions) ranged from 14.0% to 93.9%, with a lower prevalence in studies that did not apply age-related inclusion criteria. There were more female than male participants in all studies that reported sex distributions.^26,28–32,34–40^ Two studies included specialised outpatient and inpatient care data in addition to primary care data.^29,32^ The criteria for disease selection varied between studies, with chronicity of the disease being the most common criterion (n = 13),^26–29,31–35,37–39,41^ followed by high prevalence (n = 12)^28,30–40^ and clinical relevance (n = 10).^26,27,29,31,33,35,37–39,41^ The diagnostic classification tools used included the International Statistical Classification of Diseases and Related Health Problems, 10th Revision (ICD-10) and the International Classification of Primary Care, 2nd Edition (ICPC-2). Some studies^27,30,34,35^ grouped the ICD-10 and ICPC-2 codes into Expanded Diagnosis Clusters (EDCs) of the proprietary Adjusted Clinical Groups (ACG) System, while others^32,37–39,41^ used alternative disease classifications, such as those developed by Calderon-Larrañaga et al.^3^ and Barnett et al.^42^

**Table 1.** Characteristics of the included studies.

Author, year	Country	Location	Start/end year(s)	Number of primary healthcare centres	Number of individuals included in pattern identification	Multimorbidity prevalence	Age	% female	Additional data sources	Diagnostic classification tools	Disease selection criteria
Bisquera et al., 2021	United Kingdom	South London	2005-2020	41	174,882	21%	≥ 18	55%	None	Quality and Outcomes Framework and CPRD GOLD Codes List	Clinical relevance, chronic disease
Diaz et al., 2015	Norway	Norway	2008	All public primary healthcare centres nationwide	3,740,528	14%	≥ 15	NA	None	ICPC-2 diagnoses grouped according to EDC	Clinical relevance, chronic disease, impact on health services
Foguet-Boreu et al., 2015	Spain	Catalonia	2010	251	322,328	93.9%	≥ 64	57%	None	263 disease categories (blocks) based on ICD-10	Active disease, Chronic disease, High prevalence
Forslund et al., 2021	Sweden	Stockholm	2017	All primary care centers	506,611	21.6%	All ages	50%	Specialised outpatient care, inpatient care	40 chronic diseases identified as internationally clinically important using ICD-10 codes	Clinical relevance, chronic disease
García-Olmos et al., 2012	Spain	Madrid	2007	129	198,670	24.5%	≥ 14	52.3%	None	26 diseases selected by the research team from 40 chronic EDCs	High prevalence, impact on health services
Guisado-Clavero et al., 2018	Spain	Barcelona, Catalonia	2009-2014	50	190,108	92.2%	65-94	59.8%	None	ICD-10 codes converted to ICPC-2 codes before applying O’Halloran criteria	Clinical relevance, high prevalence, chronic disease
Machón et al., 2020	Spain	Gipuzkoa, Basque Country	2015-2017	Not specified	813	77% after excluding those with disability	≥ 70	55%	EHR from emergency department, specialized healthcare, hospital admission services	60 groups of chronic conditions following the classification developed by Calderón-Larrañaga et al.	High prevalence, chronic disease
Mino-León et al., 2017	Mexico	Mexico City	2013	4	38,786	50%	≥ 60	NA	None	ICD-10 codes grouped into 11 chronic disease domains	Clinical relevance, high prevalence, chronic disease
Poblador-Plou et al., 2014	The Netherlands	South of the Netherlands	2010	21	79,291	43.8%	≥ 15	51.6%	None	ICPC codes grouped into 114 chronic EDCs	High prevalence, chronic disease
Prados-Torres et al., 2012	Spain	Aragon and Catalonia	2008	19 (urban)	275,682	36.8%	≥ 15	56.6%	None	ICPC codes grouped into 114 chronic EDCs	Clinical relevance, high prevalence, chronic disease
Roso-Llorach et al., 2018 & Violan et al., 2018	Spain	Catalonia	2010	1365 general practitioners	408,944	78.1%	45-64	53.3%	None	263 disease categories using ICD-10 structure	High prevalence
Stafford et al., 2021 & Violan et al., 2020 & Violan et al., 2019	Spain	Catalonia	2012	285	916,619	93.1%	65-99	57.8%	None	ICD-10 and the 60 chronic disease categories determined by Calderón-Larrañaga et al.	Clinical relevance, chronic disease. high prevalence
Zhu et al., 2020	United Kingdom	England	2012	Not specified	113,211	29%	≥ 18	NA	None	38 LTCs based on classification of LTCs in primary care by Barnett et al.	Clinical relevance, chronic disease

Abbreviations: EDC: Expanded diagnostic cluster; EHR: Electronic health record; ICD-10: International Statistical Classification of Diseases and Related Health Problems, 10th Revision; ICPC-2: International Classification of Primary Care, 2nd Edition; LTC: Long-term conditions; NA: Not available.

### Methods and types of analysis conducted

Table 2 summarises the statistical methods used to identify multimorbidity patterns across studies. The most frequently used method was multiple correspondence analysis (MCA; n = 8),^26,30–32,37–40^ which was employed in conjunction with other methods in half of the studies that used this approach.^26,37–39^ Cluster analysis was the second most commonly used method (n = 5)^26,28,29,33,36^ and was combined with other methods in two studies.^26,36^ Four studies employed exploratory factor analysis (EFA)^27,34–36^ or principal component analysis (PCA),^33,37–39^ whereas one study used latent class analysis (LCA).^41^ There was significant variability across studies regarding aggregation methods, proximity measures, dimensionality assessment, and criteria for allocating conditions to patterns. Factor loadings were used in all studies that employed EFA, with a lower threshold of 0.25 or 0.30. An observed/expected ratio of ≥2 was used in studies that applied MCA.

**Table 2.** Statistical methods for identifying multimorbidity patterns.

Author, year	Statistical Method	Stratification variable(s)	Number of conditions included (prevalence threshold)	Aggregation method	Proximity measure	Criteria for allocating conditions to patterns	Assessment of dimensionality	Clinical interpretability of patterns
Bisquera et al., 2021	Multiple correspondence analysis and Hierarchical cluster analysis	Calendar year	32 (0%)	Ward's minimum variance	Ratio of within sums of squares to between sums of squares	Proportion (> 50%) of their conditions that belong to a cluster	Scree plot, squared cosines	Yes
Diaz et al., 2015	Exploratory factor analysis	Age, sex, and country of origin	NA (1%)	Oblique rotation (Oblimin)	Tetrachoric correlations matrices	Factor loadings ≥ 0.25	Scree plot	Yes
Foguet-Boreu et al., 2015	Cluster analysis	Age and sex	NA (1%)	Ward's minimum variance	Jaccard coefficient	NA	The highest adjusted Rand index, with a high number of clusters, and a high pseudo T2 statistic	Yes
Forslund et al., 2021	Cluster analysis	None	40 (0%)	k-means clustering	NA	NA	Cubic clustering criterion, Pseudo F and Pseudo T-Squared	Yes
García-Olmos et al., 2012	Multiple correspondence analysis	None	26 (% not listed)	Graphic technique	Category points	Absolute contributions (inertia >0.05), relative contributions, the category’s position on the axis of the dimension	Benzecri inertia adjustment	Yes
Guisado-Clavero et al., 2018	Multiple correspondence analysis	Age, sex, and calendar year	NA (1%)	k-means clustering	Jaccard coefficient	Diseases with an intra-cluster prevalence ≥ 20% and O/E ratio ≥ 2	Calinski-Harabaz criteria	Yes
Machón et al., 2020	Multiple correspondence analysis	Frailty status	50 (1%)	k-means clustering	Jaccard coefficient	NA	Balance between the number of clusters observed in the dendrogram and Calinski–Harabasz criteria	Not mentioned
Mino-León et al. 2017	Cluster analysis and principal component analysis	None	11 (% not listed)	Varimax rotation	Average linkage method, Yule’s Q	Factor loadings ≥ 0.30	Dendrogram and the agglomeration coefficient, comparative fit index, Tucker-Lewis index	Not mentioned
Poblador-Plou et al., 2014	Exploratory factor analysis	Age and sex	62 (1%)	Oblique rotation (Oblimin)	Tetrachoric correlations matrices	Factor loadings ≥ 0.25	Scree plot	Yes
Prados-Torres et al., 2012	Exploratory factor analysis	Age and sex	46 (1%)	Oblique rotation (Oblimin)	Tetrachoric correlations matrices	Factor loadings ≥ 0.25	Scree plot	Yes
Roso-Llorach et al., 2018	Hierarchical cluster analysis and exploratory factor analysis	Sex	73-79 (1%)	Ward's minimum variance, principal factors method with squared multiple correlations, Oblimin	Jaccard coefficient, Tetrachoric correlation	Factor loadings ≥ 0.30	The highest adjusted Rand index, semipartial R2, Calinski-Harabasz pseudo-F statistic and pseudo-T2 statistic criteria, Scree plot	Yes
Stafford et al., 2021	Principal component analysis and multiple correspondence analysis	None	40 (2%)	Fuzzy c-means algorithm	NA	O/E ratio ≥ 2, exclusivity ≥ 30% (secondary)	Karlis–Saporta–Spinaki rule, Fukuyama index, Xie-Beni index, Partition coefficient index, partition entropy index	Yes
Violán et al., 2018	Multiple correspondence analysis	Sex	73-79 (1%)	k-means clustering	Jaccard coefficient	O/E ratio ≥ 2	Calinski-Harabasz index	Yes
Violán et al., 2019	Principal component analysis and multiple correspondence analysis	Calendar year	49 (2%)	Fuzzy c-means algorithm	NA	O/E ratio ≥ 2, exclusivity ≥ 25%	Karlis–Saporta–Spinaki rule, Xie-Beni index, Partition coefficient index, partition entropy index	Yes
Violán et al., 2020	Principal component analysis and multiple correspondence analysis	Calendar year	47 (2%)	Fuzzy c-means algorithm	NA	O/E ratio ≥ 2	Karlis–Saporta–Spinaki rule, Xie-Beni index, Partition coefficient index, partition entropy index	Yes
Zhu et al., 2020	Latent class analysis	Age	38 (0%)	NA	NA	Qualitative assessment of O/E ratio	Bayesian Information Criteria, log-likelihood ratio test, entropy for classification quality, clinical judgment	Yes

Abbreviations: O/E: Observed/expected; NA: not available.

Four studies did not report any stratification in the identification of multimorbidity patterns,^29,30,33,37^ although Mino-León et al.^33^ only included individuals over 60, Stafford et al.^37^ included individuals aged 65-99, and García-Olmos et al.^30^ included age and sex alongside the chronic conditions in their MCA. The most common stratification variables were sex (n = 7),^27,28,31,34–36,40^ followed by age (n = 6),^27,28,31,34,35,41^ and calendar year (n = 4).^26,31,38,39^ Country of origin and frailty status were stratified for in one study each.^27,32^ Almost all studies (n = 13)^26,29,30,32–41^ reported the number of chronic conditions included in the pattern identification, with 11 of them^26,29,32,34–41^ also reporting whether any prevalence threshold was applied. Prevalence thresholds ranged from >0% to >2%. Consideration of the clinical interpretability of the generated patterns was explicitly stated in all but two studies.^32,33^

### Multimorbidity patterns

Table 3 summarises the patterns identified in each study. The following patterns repeatedly emerged as standalone or in combination with other conditions: mental health (n = 16), cardiovascular (n = 16), musculoskeletal (n = 12), respiratory (n = 11), and gastrointestinal (n = 10).

**Table 3.** Summary of multimorbidity patterns identified in the included studies.

Author, year	Additional stratification criteria (if present)	Age stratification (if present)			
Bisquera et al., 2021		**Overall**			
		Mental health; Cardiovascular; Pain; Liver disease; Dependence			
Diaz et al., 2015	**Place of birth and sex**	**15-44**	**45-64**		**65+**
	Norway, male	Mental health; Respiratory-atopic	Mental health; Cardiovascular; Cardio-endocrine; Respiratory		Mental-geriatric; Cardiovascular; Cardio-endocrine; Respiratory; Musculoskeletal; Malignant
	Norway, female	Mental health; Endocrine	Mental health; Cardio-endocrine; Respiratory		Mental-geriatric; Cardiovascular; Cardio-endocrine; Respiratory; Musculoskeletal
	West Europe & North America, male	Mental health	Mental health; Cardiovascular; Cardio-endocrine; Respiratory		Mental-geriatric; Mental health; Cardiovascular; Cardio-endocrine
	West Europe & North America, female	Mental health; Respiratory-atopic	Mental health; Cardio-endocrine		Mental-geriatric; Cardiovascular; Cardio-endocrine; Respiratory; Malignant
	Eastern Europe, male	Mental health	Mental health; Cardio-endocrine		Mental-psychosomatic; Cardiovascular; Cardio-endocrine; Malignant; Complex endocrine
	Eastern Europe, female	Mental health; Endocrine	Mental-psychiatry; Cardio-endocrine; Haematological; Musculoskeletal		Mental health; Cardio-endocrine; Haematological
	Asia, Africa and Latin America, male	Mental health; Respiratory	Mental health; Cardiovascular; Cardio-endocrine; Respiratory		Cardiovascular; Cardio-endocrine; Malignant; Musculoskeletal
	Asia, Africa and Latin America, female	Mental health; Endocrine; Haematological	Mental health; Cardio-endocrine; Endocrine; Haematological		Mental-psychosomatic; Cardiovascular; Haematological; Other (hypertension, hypothyroidism, COPD, asthma)
Foguet-Boreu et al., 2015^a^	**Sex**	**65-79**			**80+**
	Male	Cardiometabolic; Musculoskeletal; Psychiatric and respiratory; Cardiovascular			Cardiometabolic; Gastrointestinal and musculoskeletal; Cardiovascular and renal; Sensory and inflammatory
	Female	Cardiometabolic; Musculoskeletal; Complex 1; Complex 2			Cardiometabolic; Musculoskeletal; Endocrine; Gastrointestinal and metabolic
Forslund et al., 2021		**Overall**			
		Anxiety, depression, alcohol problems; Hypertension; Diabetes and hypertension; Cancer; Thyroid disorders; Hearing loss; Hypertension and cardiovascular disease			
García-Olmos et al., 2012^a^		**Overall**			
		Cardiometabolic; Cardiorenal; Psychiatric and respiratory; Complex			
Guisado-Clavero et al., 2018	**Sex and calendar year**	**65-79**			**80+**
	Male, 2009	Nonspecific; Endocrine-metabolic; Musculoskeletal; Digestive-respiratory; Neuropsychiatric; Cardiovascular			Nonspecific; Endocrine-metabolic; Musculoskeletal; Digestive-respiratory; Neuropsychiatric; Cardiovascular
	Female, 2009	Nonspecific; Musculoskeletal; Endocrine-metabolic; Digestive; Neuropsychiatric; Cardiovascular			Nonspecific; Musculoskeletal; Neuropsychiatric; Endocrine-metabolic; Cardiovascular; Digestive
	Male, 2014	Nonspecific; Endocrine-metabolic; Musculoskeletal; Digestive-respiratory; Neuropsychiatric; Cardiovascular			Nonspecific; Endocrine-metabolic; Musculoskeletal; Digestive-respiratory; Neuropsychiatric; Cardiovascular
	Female, 2014	Nonspecific; Musculoskeletal; Endocrine-metabolic; Digestive; Neuropsychiatric; Cardiovascular			Nonspecific; Musculoskeletal; Neuropsychiatric; Endocrine-metabolic; Cardiovascular; Digestive
Machón et al., 2020^a^	**Frailty status**	**Overall**			
	Robust	Nonspecific; Musculoskeletal; Cardiometabolic and renal			
	Frail	Nonspecific; Musculoskeletal; Cardiometabolic; Complex psychogeriatric and eye			
Mino-León et al., 2017	**Statistical method**	**Overall**			
	Cluster analysis	Endocrine and renal; Cardiac, respiratory, and hypertension; Psychological and neurological; Vascular, upper gastrointestinal, and musculoskeletal; Neoplasia			
	Principal component analysis	Vascular, upper gastrointestinal, and musculoskeletal; Cardiac, respiratory, and hypertension; Neoplasia; Psychological, neurological and renal; Endocrine			
Poblador-Plou et al., 2014^a^	**Sex**	**15-44**	**45-64**		**65+**
	Male	Cardiometabolic; Psychiatric and substance abuse; Musculoskeletal and depression	Cardiometabolic; Gastrointestinal, musculoskeletal and psychiatric		Cardiometabolic; Complex gastrointestinal and psychiatric
	Female	Musculoskeletal, cardio-endocrine and dermatological; Psychiatric; Neurological	Cardio-endocrine and eye; Neurological, gastrointestinal, and psychiatric		Complex cardiometabolic and neurological; Psychiatric; Complex gastrointestinal and musculoskeletal
Prados-Torres et al., 2012	**Sex**	**15-44**	**45-64**		**65+**
	Male	Cardiometabolic; Psychiatric and substance abuse	Cardiometabolic; Mechanical-obesity-thyroidal		Cardiometabolic; Psychogeriatric; Mechanical-obesity-thyroidal
	Female	Cardiometabolic; Mechanical-obesity-thyroidal	Cardiometabolic; Mechanical-obesity-thyroidal; Depressive		Cardiometabolic; Psychogeriatric; Mechanical-obesity-thyroidal; Depressive
Roso-Llorach et al., 2018^a^	**Sex**	**Overall**			
	Male, HCA	Cardiometabolic; Musculoskeletal; Psychiatric and respiratory; Sensory and dermatological			
	Male, EFA	Cardiometabolic; Cardiorenal; Psychiatric and liver; Musculoskeletal			
	Female, HCA	Musculoskeletal; Cardiometabolic; Psychiatric; Dermatological and respiratory			
	Female, EFA	Musculoskeletal; Cardiometabolic; Cardiometabolic and sensory; Respiratory and ear			
Stafford et al., 2021		**Overall**			
		Non-specific; Diabetes; Neurological and musculoskeletal, female dominant; Behavioral, neurological, and musculoskeletal (female dominant); Cardio-cerebrovascular and renal; Cardiovascular, renal, inflammatory, and respiratory; Multisystem			
Violán et al., 2018^a^	**Sex**	**Overall**			
	Male	Nonspecific; Psychiatric and liver; Gastrointestinal and genitourinary; Musculoskeletal; Cardiometabolic; Dermatological and respiratory			
	Female	Nonspecific; Musculoskeletal; Dermatological and sensory; Gastrointestinal; Cardiometabolic; Infectious			
Violán et al., 2019		**Overall**			
		Nervous and digestive; Respiratory, circulatory and nervous; Circulatory and digestive; Mental, nervous and digestive (female dominant); Mental, digestive and blood (female oldest-old dominant); Nervous, musculoskeletal and circulatory (female dominant); Genitourinary, mental, and musculoskeletal (male dominant); Non-specific (youngest-old dominant)			
Violán et al., 2020		**Overall**			
		Non-specific; Eye impairment and mental; Minority metabolic autoimmune-inflammatory; Cardio-circulatory and renal; Cardio-circulatory, mental, respiratory and genitourinary; Nervous, digestive and circulatory pattern; Respiratory and ear; Digestive; Nervous, musculoskeletal, and minor diseases; Multisystem			
Zhu et al., 2020		**18-44**	**45-64**	**65-84**	**85+**
		Depression, anxiety, pain; Pain, hearing loss, hypertension; Asthma, IBS, depression; IBS, depression, hearing loss; Psychoactive substance misuse, alcohol problems, depression	Hypertension, diabetes, pain; IBS, hearing loss, pain; Depression, pain, anxiety; Asthma, pain, COPD; Alcohol, psychoactive substance misuse, pain	Hypertension, diabetes, CKD; Hearing loss, prostate disorder, IBS; Depression, pain, anxiety; CHD, diabetes, atrial fibrillation; COPD, asthma, pain; Pain, CHD, depression	Hypertension, hearing loss, diabetes; Pain, depression, constipation; CHD, atrial fibrillation, heart failure; Asthma, COPD, pain

^a^Pattern names assigned by review authors.

Abbreviations: CHD: Coronary heart disease; CKD: Chronic kidney disease; COPD: Chronic obstructive pulmonary disease; EFA: Exploratory factor analysis; HCA: Hierarchical cluster analysis; IBS: Irritable bowel syndrome.

A mental health pattern emerged in all studies and was referred to by different names such as “mental health”, “depressive”, and “psychiatric”. This pattern was primarily characterised by depression and anxiety disorders, which were often found alongside other psychiatric diseases, neurological diseases, substance abuse disorders, gastrointestinal diseases, musculoskeletal conditions, and liver diseases. The comorbidities allocated to mental health patterns varied by age, with dementia and other ageing-related neurological conditions being more common in older individuals, whereas alcohol abuse and substance dependence being more common in younger individuals. While a mental health pattern was generally observed in most age- and sex strata, some studies did not identify it in certain subgroups. For instance, the pattern was absent among males of African, Asian, or Latin American origin aged 65 years and older in the study by Diaz et al.^27^ Similarly, among older adults aged 70 and above, Machón et al.^32^ observed a complex psychogeriatric and eye pattern in frail participants but not robust participants. Roso-Llorach et al.^36^ did not detect a psychiatric pattern using EFA but detected it through hierarchical cluster analysis (HCA).

A cardiovascular pattern was also identified in all studies. Hypertension was the most frequently reported condition within this pattern; other coexisting cardiovascular diseases included ischaemic heart disease, heart failure, or conduction disorders (e.g. atrial fibrillation). Cardiovascular conditions were often clustered with endocrine (e.g. diabetes), metabolic (e.g. lipid disorders and obesity), and renal (e.g. chronic renal failure) conditions. While Diaz et al.^27^ did not identify a cardiovascular pattern among the youngest age group (15-44), three other studies^34,35,41^ did so. Cardiovascular conditions also appeared in other patterns. For instance, Guisado-Clavero et al.^31^ reported cerebrovascular diseases (e.g. stroke) as part of a neuropsychiatric pattern, whereas Prados-Torres et al.^35^ identified cardiovascular diseases within a psychogeriatric pattern among females aged 65 years and older. García-Olmos et al.^30^ reported cardiac valve diseases and generalised atherosclerosis as part of a complex pattern that included several conditions across multiple organ systems.

The musculoskeletal pattern, also called the mechanical pattern, was consistently identified across age and sex strata in twelve studies^27,28,31–40^, irrespective of frailty status and the statistical method used. Among others, this pattern included broader disease groups (e.g. arthropathies, dorsopathies, soft tissue diseases) and specific conditions and/or symptoms (e.g. osteoarthritis, lower back pain, cervical pain). Such patterns were identified as standalone or coupled with other conditions, such as psychiatric, cardiovascular, neurological, and gastrointestinal diseases. Two studies identified female-dominant clusters in which musculoskeletal conditions were coupled with neurological conditions (e.g. peripheral neuropathy).^37,39^ In contrast, in a male-dominant cluster, these conditions were coupled with genitourinary and mental health conditions.^39^

Respiratory patterns encompassed a range of upper and lower respiratory tract diseases, such as asthma, chronic obstructive pulmonary disease (COPD), viral infections, and nose and throat conditions. These conditions were often reported in conjunction with pain and other diseases affecting different organ systems, such as the dermatological, cardiovascular, gastrointestinal, renal, genitourinary, and nervous systems. Diaz et al.^27^ found a respiratory pattern across all age groups and both sexes, but the identification and composition of the pattern varied depending on the participants’ country of origin. For instance, a respiratory pattern was absent among individuals from Eastern Europe and appeared as part of a larger pattern with hypertension and hypothyroidism among older females from Asia, Africa, and Latin America. Foguet-Boreu et al.^28^ identified a respiratory pattern in the youngest-old (aged 65-79) but not in the oldest-old group (aged 80 years and above). In the latter study, chronic lower respiratory diseases were coupled with psychiatric conditions in a standalone pattern in males. In contrast, in females, respiratory conditions were part of two larger, complex patterns. Guisado-Clavero et al.^31^ did not identify a respiratory pattern among Spanish females aged 65 and above, while Roso-Llorach et al.^36^ did detect such a pattern among both Spanish males and females aged 45-64, albeit the pattern failed to emerge in males using EFA as the statistical method. Finally, Zhu et al.^41^ found respiratory conditions coupled with irritable bowel syndrome and depression among younger participants (aged 18-44), and with pain among participants of all other ages (45 and above).

A gastrointestinal pattern was reported in ten studies^26,28,31,33,34,36,38–41^ and included liver diseases, cholelythiasis, gastroesophageal reflux disease, diverticulitis of the colon, and other diseases of the oesophagus, stomach, and intestines. These diseases mostly formed complex patterns with other diseases, such as neurological, psychiatric, and metabolic, and less frequently with genitourinary, haematological, cardiovascular, and musculoskeletal diseases. Bisquera et al.^26^ identified a standalone liver disease pattern, whereas Roso-Llorach et al.^36^ identified a pattern grouping psychiatric and liver diseases, but only in men. Zhu et al.^41^ grouped irritable bowel syndrome into several different patterns among those aged between 18-84. Prados-Torres et al.^35^ found gastrointestinal diseases as part of the mechanical-obesity-thyroid pattern, similar to Mino-León et al.,^33^ who found upper gastrointestinal conditions coupled with musculoskeletal and vascular disorders among Mexicans aged 60 years and above.

Other patterns identified in a limited number of studies were sensory (including eye and ear conditions; n = 7),^28,29,32,34,36,38,40^ genitourinary (n = 4),^38–41^ dermatological (n = 3),^34,36,40^ and malignant (n = 3).^27,29,33^ Several studies also identified complex^28,30,34,37,38^ and non-specific^31,32,37–40^ clusters, which had either multiple or no overrepresented conditions or organ systems, respectively. Further information regarding these patterns is available in the Supplementary Excel File.

## Discussion

This systematic review of 16 studies investigated multimorbidity patterns in primary care settings using EHR data. The majority of the studies were conducted in Spain and included participants of all ages and sexes. Significant heterogeneity in clustering methods and disease classification tools challenged the synthesis of the results; however, mental health and cardiovascular patterns were identified in all studies. Three other patterns containing musculoskeletal, respiratory, and gastrointestinal diseases, respectively, were also found in most studies.

### Identified patterns

The identification of a mental health pattern in all studies included in this review is in line with findings from previous reviews, where mental health patterns emerged across all populations and statistical approaches, adding weight to the evidence that such patterns are not coincidental.^20,21^ Due to their chronic and recurrent nature, mental health conditions have a significant impact on individuals and healthcare systems alike, and may point to important avenues for intervention.^43^ The association of mental health conditions with other diseases, such as gastrointestinal and musculoskeletal conditions, may be explained by shared pathophysiological mechanisms, such as chronic mild inflammation,^44^ oxidative stress,^45^ and altered gut-brain axis communication.^46^ There is also evidence that mental health conditions exacerbate pre-existing physical conditions, or vice versa, highlighting the need for integrated approaches to care.^47^

Similarly, a cardiovascular pattern emerged in all studies included in the review. This finding is also consistent with those of previous reviews.^10,20,21^ The presence of cardiovascular conditions in a variety of patterns highlights the complex interplay between the cardiovascular system and other organ systems. Early detection and effective management of hypertension, a common precursor to other cardiovascular, endocrine, and renal conditions,^48^ may prevent or delay the transition of individuals with hypertension into more complex cardiometabolic and cardio-endocrine clusters. The coexistence of cerebrovascular diseases in the neuropsychiatric pattern corroborates the close link between the neurological and cardiovascular systems.^49^ Additionally, the presence of cardiovascular diseases within psychogeriatric patterns supports the need to perform a comprehensive geriatric assessment among older adults, and to reinforce the collaboration between medical and psychiatric care providers.

Some patterns, such as the ones characterised by musculoskeletal, respiratory, and gastrointestinal diseases, were identified in some, but not all, studies. The mechanisms underlying these patterns are likely multifactorial, and could include genetic, environmental, lifestyle, and demographic factors.^50^ For example, previously identified sex differences in hormonal and anatomical systems, physical activity levels, and/or reporting of pain could explain the clustering of musculoskeletal conditions in female-dominant patterns.^51^ Differences in environmental and infectious exposures^52,53^ and healthcare access and utilisation^54^ could explain the variation in respiratory patterns across countries of birth observed in the study by Diaz et al.,^27^ where a respiratory pattern was observed among participants born in all parts of the world, except in Eastern Europe. The coexistence of gastrointestinal conditions with neurological and metabolic conditions may be related to inflammatory and immunological dysregulation through shared risk factors (e.g. unhealthy diet and obesity) and gut microbiome dysbiosis,^46,55^ among others.

It is not surprising that other patterns, such as those containing dermatological or malignant diseases, were only observed in a subset of studies. Chronic conditions can be influenced by a variety of factors, such as socioeconomic and lifestyle factors, genetic predisposition, health literacy level and healthcare-seeking behaviours, which can vary greatly across populations. These variations can lead to differences in the development and prognosis of chronic conditions, as reflected in the heterogeneity of multimorbidity patterns identified in this review. However, determining the extent to which these findings are truly due to variations in the aforementioned factors rather than methodological differences between studies is challenging.

### Impact of methodology

Most studies included in this systematic review were of high quality, largely owing to the use of primary care EHRs as the data source. Primary care serves as the entry point into healthcare services, particularly for marginalised and underserved population groups.^56^ The significance of primary care for multimorbidity research is further strengthened by its person-centred nature, capturing physical, mental, and social aspects of health throughout the life course and across medical specialties.^56^ Moreover, the population-wide coverage of primary care EHR data ensures the completeness of information and enables the analysis of large sample sizes that more accurately represent target populations.^18^ Leveraging these inherent strengths of primary care EHRs lays a solid foundation for exploring multimorbidity patterns in diverse populations. However, several methodological challenges remain to be considered (Table 4).

**Table 4.** Methodological limitations and solutions in studying multimorbidity patterns in primary care.

Methodological limitation	Problem	Solution
Lack of adjustment/stratification for sociodemographic factors (e.g. age and/or sex)	Heterogeneity and correlation between chronic conditions and sociodemographic factors may be obscured in non-stratified analyses	Use large datasets with sufficient sample size and information on relevant adjustment/stratification factors Explore sociodemographic characteristics of patterns obtained from non-stratified analyses
Accuracy and comprehensiveness of diagnoses	Missing or incomplete diagnoses underestimate chronic disease burden	Supplement primary care data with specialized outpatient, inpatient, prescription or other relevant data
Quality and consistency of data recorded	Same conditions may be coded differently due to variations in coding practices across general practitioners	Group similar codes into broader diagnostic groups to reduce disease misclassification, while considering the potential loss of disease specificity
Lack of systematicity in identification of multimorbidity patterns	Limited comparability of findings due to significant heterogeneity in clustering techniques and identification of overexpressed conditions	Develop consensus on clustering techniques and criteria for identifying overexpressed conditions
Reporting and naming patterns	Lack of clarity and meaningful interpretation	Strive for balanced cluster names that capture the essence of findings without oversimplifying or omitting important information

Most articles only partially met the quality criterion related to stratification and/or adjustment for sociodemographic and other relevant factors. Sociodemographic factors, such as age, sex, and socioeconomic status, are known to be strongly correlated with the incidence and interplay between chronic conditions.^57^ For example, in another review, cardiometabolic patterns were more common among men of lower socioeconomic status, whereas musculoskeletal patterns were more common among women.^57^ Clustering algorithms may capture some of this heterogeneity in the absence of stratification; therefore, researchers should carefully explore the sociodemographic characteristics of the patterns obtained from non-stratified analyses as done in a paper included in this review, which identified "female-dominant" or "youngest-old dominant" patterns in the total population.^39^ The only study that managed to meet all the quality criteria was by Diaz et al.,^27^ which stratified the entire Norwegian population by age group and country of birth and found considerable differences in pattern composition across different strata. Although small sample sizes may hinder stratification, most reviewed studies included more than 100,000 participants. Therefore, we emphasise the importance of this quality criterion for researchers working with such large datasets. This approach can identify unique patterns and associations that might be obscured or diluted in total population analyses, ultimately offering novel insights into the pathophysiology and complexity of multimorbidity and contributing to better-informed decision-making by healthcare providers and policymakers.

The identification of multimorbidity patterns is a complex task that relies heavily on the accurate and comprehensive detection of individual conditions, which, despite being expectedly higher in primary care EHR-based studies, could also be threatened by several factors. First, missing or incomplete diagnoses for individuals who sought care at non-participating health centres (especially in places where care continuity is suboptimal) or whose symptoms did not require a visit to a physician might result in an underestimation of the prevalence of certain conditions.^18^ Some studies included in the review supplemented primary care data with specialised outpatient, inpatient, or prescription data, which may help reduce misclassification when population coverage is expected to be an issue, resulting in more accurate identification of multimorbidity patterns. However, these additional sources were not always utilized in the ascertainment of chronic conditions by the studies included in the review. We recommend that researchers make use of all available diagnostic data sources whenever their primary objective is not to explore one particular data source, but instead to gain a thorough understanding of the participants’ multimorbidity profiles.

Another challenge that accompanies the use of EHR data relates to the quality and consistency of the data recorded by different primary care providers. Indeed, the accuracy of diagnoses can vary widely among general practitioners due to differences in diagnosis coding practices.^18^ To address this issue, some studies have grouped similar ICD-10 codes into Expanded Diagnostic Clusters (EDC) or even higher-level classifications, such as that developed by Calderon-Larrañaga et al.,^3^ which groups all chronic ICD-10 codes into 60 groups based on shared pathophysiology. Such classifications may result in lower misclassification rates, albeit at the expense of information loss regarding disease specificity, staging, and severity. This approach may be suitable for disease-centred cluster analyses that aim to identify the common aetiology of conditions; however, it may not be well suited for analyses that aim to identify groups of individuals with similar disease patterns. In such analyses, a disease may belong to more than one pattern, and the use of higher-level categories may hinder the identification of individuals who could benefit from earlier and/or more targeted interventions. For instance, Sullivan et al.^58^ found that the composition of multimorbidity patterns varied significantly based on the level of kidney dysfunction assessed using the estimated glomerular filtration rate (eGFR), with cardiovascular conditions becoming increasingly prominent at lower eGFR levels. Therefore, careful consideration of accuracy and granularity when defining chronic conditions is important to maintain a balance between disease misclassification and loss of potentially critical information.

Finally, the lack of consensus and methodological alignment and harmonisation in identifying and naming multimorbidity patterns poses a significant challenge to understanding the complex relationships between chronic conditions and developing appropriate management strategies.^10^ Inconsistencies in the application of clustering techniques and identification of overexpressed diseases result in variations in the number, size, and composition of the identified patterns, making it difficult to compare findings and draw meaningful conclusions.^10^ Furthermore, it is important to note that the process of reporting and naming patterns presents challenges in itself. In some cases, cluster names may become overly simplistic, leading to loss of important information. Conversely, names that are too broad may list all the conditions comprising the pattern without specifically identifying the overexpressed or leading conditions. Striking the right balance when reporting patterns is crucial to accurately capture the essence of the findings while ensuring clarity and meaningful interpretation.

### Strengths and limitations

The strengths of this review include the quality assessment of included studies and its explicit focus on EHRs from primary care as the data source, addressing the heterogeneity in data sources and disease classification highlighted in previous reviews. A limitation of our review is the lack of protocol registration. The generalisability of our findings may be limited, as most of the studies included in this review were from Europe, which suggests that access to EHR data remains a challenge in many other parts of the world. Nevertheless, existing studies can still provide valuable insights into the epidemiology of multimorbidity for other regions as well. As access to diverse and high-quality data sources continues to improve, we can expect to gain a more nuanced understanding of multimorbidity patterns and their determinants across different populations in the coming years.

## Conclusions

This systematic review examined and synthesised multimorbidity patterns in primary care settings across 16 studies. Despite considerable methodological differences among the studies, several consistent patterns emerged. Mental health and cardiovascular patterns were identified in all studies, while patterns containing musculoskeletal, respiratory, and gastrointestinal diseases were identified in the majority of studies. These findings contribute to the growing body of evidence on replicable multimorbidity patterns and highlight the importance of integrated care approaches that consider the complex interactions between physical and mental health conditions. Further research is needed to gain a deeper understanding of the underlying mechanisms and develop targeted preventive primary care and public health interventions.

## Supplemental Material

Supplemental Material - Patterns of multimorbidity in primary care electronic health records: A systematic review Click here for additional data file. Supplemental Material for Patterns of multimorbidity in primary care electronic health records: A systematic review by Giorgi Beridze, Ahmad Abbadi, Joan Ars, Francesca Remelli, Davide L Vetrano, Caterina Trevisan, Laura-Mónica Pérez, Juan A López-Rodríguez, and Amaia Calderón-Larrañaga in Journal of Multimorbidity and Comorbidity

Supplemental Material - Patterns of multimorbidity in primary care electronic health records: A systematic review Click here for additional data file. Supplemental Material for Patterns of multimorbidity in primary care electronic health records: A systematic review by Giorgi Beridze, Ahmad Abbadi, Joan Ars, Francesca Remelli, Davide L Vetrano, Caterina Trevisan, Laura-Mónica Pérez, Juan A López-Rodríguez, and Amaia Calderón-Larrañaga in Journal of Multimorbidity and Comorbidity
