# Supplementary material for: Patterns of multimorbidity in primary care electronic health records: A systematic review
Source: J Multimorb Comorb. 2024 Jan 30;14:26335565231223350. doi: 10.1177/26335565231223350 (PMC10829499; doi:10.1177/26335565231223350)
Supplement: Supplemental Material - Patterns of multimorbidity in primary care electronic health records: A systematic review [file sj-pdf-2-cob-10.1177_26335565231223350.pdf]

Supplementary Table 1. Preferred Reporting Items for Systematic Reviews and Meta-Analyses (PRISMA) 2020 checklist 2

Supplementary Table 2A. Search strategy in MEDLINE 5

Supplementary Table 2B. Search strategy in Web of Science Core Collection 6

Supplementary Table 2C. Search strategy in Cinahl. 7

Supplementary Box. Modified Newcastle-Ottawa Quality Assessment Scale (Adapted for cross-sectional studies) 8

Supplementary Table 3. List of excluded studies at full-text review and first reason for exclusion 9

Supplementary Table 4. Quality assessment of the included studies. 13

Supplementary Table 1. Preferred Reporting Items for Systematic Reviews and Meta-Analyses (PRISMA) 2020 checklist

Section and Topic	Item #	Checklist item	Location where item is reported
**TITLE**			
Title	1	Identify the report as a systematic review.	Page 1
**ABSTRACT**			
Abstract	2	See the PRISMA 2020 for Abstracts checklist.	Page 2
**INTRODUCTION**			
Rationale	3	Describe the rationale for the review in the context of existing knowledge.	Page 3
Objectives	4	Provide an explicit statement of the objective(s) or question(s) the review addresses.	Page 4
**METHODS**			
Eligibility criteria	5	Specify the inclusion and exclusion criteria for the review and how studies were grouped for the syntheses.	Pages 4-5
Information sources	6	Specify all databases, registers, websites, organisations, reference lists and other sources searched or consulted to identify studies. Specify the date when each source was last searched or consulted.	Page 4
Search strategy	7	Present the full search strategies for all databases, registers and websites, including any filters and limits used.	Page 4 and supplementary material
Selection process	8	Specify the methods used to decide whether a study met the inclusion criteria of the review, including how many reviewers screened each record and each report retrieved, whether they worked independently, and if applicable, details of automation tools used in the process.	Page 5
Data collection process	9	Specify the methods used to collect data from reports, including how many reviewers collected data from each report, whether they worked independently, any processes for obtaining or confirming data from study investigators, and if applicable, details of automation tools used in the process.	Page 5
Data items	10a	List and define all outcomes for which data were sought. Specify whether all results that were compatible with each outcome domain in each study were sought (e.g. for all measures, time points, analyses), and if not, the methods used to decide which results to collect.	N/A
	10b	List and define all other variables for which data were sought (e.g. participant and intervention characteristics, funding sources). Describe any assumptions made about any missing or unclear information.	Page 5
Study risk of bias assessment	11	Specify the methods used to assess risk of bias in the included studies, including details of the tool(s) used, how many reviewers assessed each study and whether they worked independently, and if applicable, details of automation tools used in the process.	Pages 5-6
Effect measures	12	Specify for each outcome the effect measure(s) (e.g. risk ratio, mean difference) used in the synthesis or presentation of results.	N/A
Synthesis methods	13a	Describe the processes used to decide which studies were eligible for each synthesis (e.g. tabulating the study intervention characteristics and comparing against the planned groups for each synthesis (item #5)).	Page 6
	13b	Describe any methods required to prepare the data for presentation or synthesis, such as handling of missing summary statistics, or data conversions.	N/A

Section and Topic	Item #	Checklist item	Location where item is reported
	13c	Describe any methods used to tabulate or visually display results of individual studies and syntheses.	Page 6
	13d	Describe any methods used to synthesize results and provide a rationale for the choice(s). If meta-analysis was performed, describe the model(s), method(s) to identify the presence and extent of statistical heterogeneity, and software package(s) used.	Page 6
	13e	Describe any methods used to explore possible causes of heterogeneity among study results (e.g. subgroup analysis, meta-regression).	N/A
	13f	Describe any sensitivity analyses conducted to assess robustness of the synthesized results.	N/A
Reporting bias assessment	14	Describe any methods used to assess risk of bias due to missing results in a synthesis (arising from reporting biases).	Page 5 and supplementary material
Certainty assessment	15	Describe any methods used to assess certainty (or confidence) in the body of evidence for an outcome.	Page 5 and supplementary material
**RESULTS**			
Study selection	16a	Describe the results of the search and selection process, from the number of records identified in the search to the number of studies included in the review, ideally using a flow diagram.	Page 6
	16b	Cite studies that might appear to meet the inclusion criteria, but which were excluded, and explain why they were excluded.	Supplementary material
Study characteristics	17	Cite each included study and present its characteristics.	Pages 6-7
Risk of bias in studies	18	Present assessments of risk of bias for each included study.	Page 6 and supplementary material
Results of individual studies	19	For all outcomes, present, for each study: (a) summary statistics for each group (where appropriate) and (b) an effect estimate and its precision (e.g. confidence/credible interval), ideally using structured tables or plots.	Page 8 and table 3
Results of syntheses	20a	For each synthesis, briefly summarise the characteristics and risk of bias among contributing studies.	Pages 8-10
	20b	Present results of all statistical syntheses conducted. If meta-analysis was done, present for each the summary estimate and its precision (e.g. confidence/credible interval) and measures of statistical heterogeneity. If comparing groups, describe the direction of the effect.	Pages 8-10
	20c	Present results of all investigations of possible causes of heterogeneity among study results.	N/A
	20d	Present results of all sensitivity analyses conducted to assess the robustness of the synthesized results.	N/A
Reporting biases	21	Present assessments of risk of bias due to missing results (arising from reporting biases) for each synthesis assessed.	N/A
Certainty of evidence	22	Present assessments of certainty (or confidence) in the body of evidence for each outcome assessed.	N/A
**DISCUSSION**			

Section and Topic	Item #	Checklist item	Location where item is reported
Discussion	23a	Provide a general interpretation of the results in the context of other evidence.	Pages 10-11
	23b	Discuss any limitations of the evidence included in the review.	Pages 11-12
	23c	Discuss any limitations of the review processes used.	Page 13
	23d	Discuss implications of the results for practice, policy, and future research.	Pages 11-13
**OTHER INFORMATION**			
Registration and protocol	24a	Provide registration information for the review, including register name and registration number, or state that the review was not registered.	13
	24b	Indicate where the review protocol can be accessed, or state that a protocol was not prepared.	N/A
	24c	Describe and explain any amendments to information provided at registration or in the protocol.	N/A
Support	25	Describe sources of financial or non-financial support for the review, and the role of the funders or sponsors in the review.	13
Competing interests	26	Declare any competing interests of review authors.	13
Availability of data, code and other materials	27	Report which of the following are publicly available and where they can be found: template data collection forms; data extracted from included studies; data used for all analyses; analytic code; any other materials used in the review.	N/A

From: Page MJ, McKenzie JE, Bossuyt PM, Boutron I, Hoffmann TC, Mulrow CD, et al. The PRISMA 2020 statement: an updated guideline for reporting systematic reviews. BMJ 2021;372:n71. doi: 10.1136/bmj.n71

For more information, visit: http://www.prisma-statement.org/

Supplementary Table 2A. Search strategy in MEDLINE

Interface: Ovid MEDLINE(R) and Epub Ahead of Print, In-Process & Other Non-Indexed Citations and Daily

Date of Search: 26 April 2022

Number of hits: 1,792

Comment: In Ovid, two or more words are automatically searched as phrases; i.e. no quotation marks are needed

Field labels

- exp/ = exploded MeSH term
- / = non exploded MeSH term
- .ti,ab,kf. = title, abstract and author keywords
- adjx = within x words, regardless of order
- * = truncation of word for alternate endings

Database(s): **Ovid MEDLINE(R) and Epub Ahead of Print, In-Process, In-Data-Review & Other Non-Indexed Citations and Daily** 1946 to April 25, 2022
Search Strategy:

#	Searches	Results
1	exp Comorbidity/	123621
2	Multiple chronic conditions/	636
3	(co-morbid* or comorbid* or multi-condition* or multi-morbid* or multicondition* or multimorbid*).ti,ab,kf.	233797
4	((co-exist* or co-occurr* or coexist* or combin* or con-current* or concurrent* or cooccur* or multiple) adj4 (condition* or diagnos* or disease* or health problem* or illness*)).ti,ab,kf.	131386
5	or/1-4	425838
6	(pattern* or cluster*).ti,ab,kf.	1791323
7	5 and 6	30117
8	Primary Health Care/	87671
9	exp General Practice/	77377
10	(general practice* or family practice* or primary care or primary healthcare or primary health care).ti,ab,kf.	202428
11	Electronic health records/	25003
12	((computeri#ed or electronic* or digital) adj2 (medical record? or health record?)).ti,ab,kf.	46377
13	or/8-12	312590
14	7 and 13	1826
15	limit 14 to english language	1796
16	(congress or clinical conference or comment or editorial or letter).pt.	2128336
17	15 not 16	1792

Supplementary Table 2B. Search strategy in Web of Science Core Collection

Interface: Clarivate Analytics

Editions = A&HCI , ESCI , SCI-EXPANDED , SSCI

Date of Search: 26 April 2022

Number of hits: 2,187

Field labels

- TS/Topic = title, abstract, author keywords and Keywords Plus
- NEAR/x = within x words, regardless of order
- * = truncation of word for alternate endings

Note: the *Exact search*-function was used for all the searches

11
#8 AND #5 and English (Languages) and Proceedings Papers or Meeting Abstracts or Editorial Materials (Exclude – Document Types)
2,187

10
#8 AND #5 and English (Languages)
2,257

9
#8 AND #5
2,295

8
#7 OR #6
247,122

7
TS=((computeri?ed or electronic* or digital) NEAR/2 ("medical record$" or "health record$"))
43,663

6
TS=("general practice*" or "family practice*" or "primary care" or "primary healthcare" or "primary health care")
209,810

5
#4 AND #3
35,598

4
TS=(pattern* or cluster*)
3,362,198

3
#2 OR #1
418,920

2
TS=(("co-exist*" or "co-occurr*" or coexist* or combin* or "con-current*" or concurrent* or cooccur* or multiple) NEAR/4 (condition* or diagnos* or disease* or "health problem*" or illness*))
170,298

1
TS=("co-morbid*" or comorbid* or "multi-condition*" or "multi-morbid*" or multicondition* or multimorbid*)
257,489

Supplementary Table 2C. Search strategy in Cinahl.

Interface: Ebsco

Date of Search: 26 April 2022

Number of hits: 851

Field labels

- MH+ = exploded Cinahl Heading
- MH = non exploded Cinahl Heading
- TI = title
- AB = abstract
- Nx = within x words, regardless of order
- * = truncation of word for alternate endings

#	Query	Results
S14	S6 AND S12<br><br>Narrow by Language: - english	851
S13	S6 AND S12	852
S12	S7 OR S8 OR S9 OR S10 OR S11	185,386
S11	TI (((computeri?ed or electronic* or digital) N2 ("medical record#" or "health record#"))) OR AB (((computeri?ed or electronic* or digital) N2 ("medical record#" or "health record#")))	25,791
S10	(MH "Electronic Health Records")	28,167
S9	TI ("general practice*" or "family practice*" or "primary care" or "primary healthcare" or "primary health care") OR AB ("general practice*" or "family practice*" or "primary care" or "primary healthcare" or "primary health care")	106,759
S8	(MH "Family Practice")	26,416
S7	(MH "Primary Health Care")	70,452
S6	S4 AND S5	10,060
S5	TI (pattern* or cluster*) OR AB (pattern* or cluster*)	245,839
S4	S1 OR S2 OR S3	153,507
S3	TI (("co-exist*" or "co-occurr*" or coexist* or combin* or "con-current*" or concurrent* or cooccur* or multiple) N4 (condition* or diagnos* or disease* or "health problem*" or illness*)) OR AB (("co-exist*" or "co-occurr*" or coexist* or combin* or "con-current*" or concurrent* or cooccur* or multiple) N4 (condition* or diagnos* or disease* or "health problem*" or illness*))	33,729
S2	TI ("co-morbid*" or comorbid* or "multi-condition*" or "multi-morbid*" or multicondition* or multimorbid*) OR AB ("co-morbid*" or comorbid* or "multi-condition*" or "multi-morbid*" or multicondition* or multimorbid*)	83,625
S1	(MH "Comorbidity")	68,118

Supplementary Box. Modified Newcastle-Ottawa Quality Assessment Scale (Adapted for cross-sectional studies)

- **Selection: (maximum 5 stars)**

1) Representativeness of the sample:
   a. Truly representative of the average in the target population: all primary care centers in that area or a random sample of them (1 star*)
   b. Somewhat representative of the average in the target population: several primary care centers, trying to capture the socio-economic diversity of the area (1 star*)
   c. Selected group of users
   d. No description of the sampling strategy
2) Sample size:
   a. Justified and satisfactory: arbitrary choice of 1000-2000 minimum patients (1 star*)
   b. Not justified
3) Non-respondents:
   a. Comparability between respondents and non-respondents' characteristics is established, and the response rate is satisfactory (1 star*)
   b. The response rate is unsatisfactory, or the comparability between respondents and non-respondents is unsatisfactory
   c. No description of the response rate or the characteristics of the responders and the non-responders
4) Ascertainment of the exposure (risk factor):
   a. Validated measurement tool: either run quality assessment of the primary care diagnostic data or refer to other publications where the dataset has been used (2 stars**)
   b. Non-validated measurement tool, but the tool is available or described: papers which have used primary care diagnostic data without any mention of the quality check (1 star*)
   c. No description of the measurement tool

- **Comparability: (maximum 2 stars)**

1) The subjects in different outcome groups are comparable, based on the study design or analysis. Confounding factors are controlled
   a. The study controls for the most important factor: stratification or adjustment by age and sex (1 star*)
   b. The study control for any additional factor: stratification or adjustment by socio-economic status, frailty, education, ethnicity, etc. (1 star*)

- **Outcome: (Maximum 1 star)**

1) Statistical test:
   a. The statistical test used to analyze the data is clearly described and appropriate, and the measurement of the association is presented: proximity measures, type of clustering algorithm, pattern number determination, etc. (1 star*)
   b. The statistical test is not appropriate, not described or incomplete

Note: Outcome point on assessment of the outcome was removed as there were no outcomes to be studied in our research question. Therefore, maximum stars can be allocated to a study is 8 stars.

Supplementary Table 3. List of excluded studies at full-text review and first reason for exclusion

Author	Year	First Reason for Exclusion
Abad-Diez et al. [1]	2014	Studies that did not derive original patterns
Arnold-Reed et al. [2]	2018	Does not explicitly describe the method(s) used for exploring multimorbidity patterns
Bekic et al. [3]	2019	Not based on populations and electronic data from primary care
Brett et al. [4]	2013	Does not focus on the identification of patterns of associative multimorbidity
Britt et al. [5]	2008	Not based on populations and electronic data from primary care
Buja et al. [6]	2018	Not based on populations and electronic data from primary care
Carmona-Pirez et al. [7]	2022	Not based on populations and electronic data from primary care
Collerton et al.[8]	2016	Not based on populations and electronic data from primary care
Deruaz-Luyet et al.[9]	2017	Does not explicitly describe the method(s) used for exploring multimorbidity patterns
Dorrington et al. [10]	2020	Does not explicitly describe the method(s) used for exploring multimorbidity patterns
Egan et al. [11]	2019	Not based on populations and electronic data from primary care
Freund et al. [12]	2012	Does not focus on the identification of patterns of associative multimorbidity
Guo et al. [13]	2021	Not based on populations and electronic data from primary care
Hassaine et al. [14]	2020	Not based on populations and electronic data from primary care
Hunter et al. [15]	2021	Not based on populations and electronic data from primary care
Ibarra-Castillo et al. [16]	2018	Studies that did not derive original patterns
Lenzi et al. [17]	2016	Not based on populations and electronic data from primary care
Li et al. [18]	2016	Not based on populations and electronic data from primary care
Linden et al. [19]	2022	Does not explicitly describe the method(s) used for exploring multimorbidity patterns
Mohideen et al. [20]	2021	Does not focus on the identification of patterns of associative multimorbidity
Mujica-Mota et al. [21]	2015	Not based on populations and electronic data from primary care
Newcomer et al. [22]	2011	Not based on populations and electronic data from primary care
Nicholson et al. [23]	2019	Does not focus on the identification of patterns of associative multimorbidity
Pati et al. [24]	2019	Does not focus on the identification of patterns of associative multimorbidity
Pati et al. [25]	2017	Not based on populations and electronic data from primary care

Pefoyo et al. [26]	2015	Not based on populations and electronic data from primary care
Pengpid et al. [27]	2017	Does not explicitly describe the method(s) used for exploring multimorbidity patterns
Prazeres et al. [28]	2015	Not based on populations and electronic data from primary care
Quinones et al. [29]	2021	Does not focus on the identification of patterns of associative multimorbidity
Saltman et al. [30]	2005	Does not explicitly describe the method(s) used for exploring multimorbidity patterns
Shi et al. [31]	2021	Does not focus on the identification of patterns of associative multimorbidity
Singh et al. [32]	2019	Does not focus on the identification of patterns of associative multimorbidity
Sinnige et al. [33]	2015	Does not focus on the identification of patterns of associative multimorbidity
Soley-Bori et al. [34]	2022	Does not focus on the identification of patterns of associative multimorbidity
Tan et al. [35]	2020	Not based on populations and electronic data from primary care
van den Akker et al. [36]	1998	Does not focus on the identification of patterns of associative multimorbidity
van Oostrom et al. [37]	2012	Does not explicitly describe the method(s) used for exploring multimorbidity patterns
Vargese et al. [38]	2020	Does not focus on the identification of patterns of associative multimorbidity
Villen et al. [39]	2020	Studies that did not derive original patterns
Violan et al. [40]	2014	Does not focus on the identification of patterns of associative multimorbidity
Vos et al. [41]	2015	Does not explicitly describe the method(s) used for exploring multimorbidity patterns
Walker et al. [42]	2016	Not based on populations and electronic data from primary care
Wong et al. [43]	2017	Does not focus on the identification of patterns of associative multimorbidity
Yang et al. [44]	2021	Begin with a preliminary selection of index disease
Zhang et al. [45]	2019	Not based on populations and electronic data from primary care

References

1. Abad-Díez JM, Calderón-Larrañaga A, Poncel-Falcó A, et al. Age and gender differences in the prevalence and patterns of multimorbidity in the older population. *BMC Geriatr*. 2014;14(1):75. doi:10.1186/1471-2318-14-75

2. Arnold-Reed D, Troeung L, Brett T, et al. Increasing multimorbidity in an Australian street health service: A 10-year retrospective cohort study. *Australian Journal Of General Practice*. 2018;47(4):181-189. doi:10.31128/AFP-10-17-4370

3. Bekic S, Babic F, Filipcic I, Trtica Majnaric L. Clustering of Mental and Physical Comorbidity and the Risk of Frailty in Patients Aged 60 Years or More in Primary Care. *Medical Science Monitor*. 2019;25:6820-6835. doi:10.12659/MSM.915063

4. Brett T, Arnold-Reed DE, Popescu A, et al. Multimorbidity in patients attending 2 Australian primary care practices. *Annals of Family Medicine*. 2013;11(6):535-542. doi:10.1370/afm.1570

5. Britt HC, Harrison CM, Miller GC, Knox SA. Prevalence and patterns of multimorbidity in Australia. *Medical Journal of Australia*. 2008;189(2):72-77.

6. Buja A, Claus M, Perin L, et al. Multimorbidity patterns in high-need, high-cost elderly patients. *PLoS ONE [Electronic Resource]*. 2018;13(12):e0208875. doi:10.1371/journal.pone.0208875
7. Carmona-Pírez J, Poblador-Plou B, Poncel-Falcó A, et al. Applying the FAIR4Health Solution to Identify Multimorbidity Patterns and Their Association with Mortality through a Frequent Pattern Growth Association Algorithm. *IJERPH*. 2022;19(4):2040. doi:10.3390/ijerph19042040
8. Collerton J, Jagger C, Yadegarfar ME, et al. Deconstructing Complex Multimorbidity in the Very Old: Findings from the Newcastle 85+ Study. *BioMed Research International*. 2016;2016:8745670. doi:10.1155/2016/8745670
9. Deruaz-Luyet A, N'Goran AA, Senn N, et al. Multimorbidity and patterns of chronic conditions in a primary care population in Switzerland: a cross-sectional study. *BMJ Open*. 2017;7(6):e013664. doi:10.1136/bmjopen-2016-013664
10. Dorrington S, Carr E, Stevelink SAM, et al. Multimorbidity and fit note receipt in working-age adults with long-term health conditions. *Psychological Medicine*. Published online 2020:1-10. doi:10.1017/S0033291720002937
11. Egan BM, Sutherland SE, Tilkemeier PL, Davis RA, Rutledge V, Sinopoli A. A cluster-based approach for integrating clinical management of Medicare beneficiaries with multiple chronic conditions. *PLoS ONE [Electronic Resource]*. 2019;14(6):e0217696. doi:10.1371/journal.pone.0217696
12. Freund T, Kunz CU, Ose D, Szecsenyi J, Peters-Klimm F. Patterns of multimorbidity in primary care patients at high risk of future hospitalization. *Population Health Management*. 2012;15(2):119-124. doi:10.1089/pop.2011.0026
13. Guo XR, Zhao BH, Chen TM, Hao B, Yang T, Xu HM. Multimorbidity in the elderly in China based on the China Health and Retirement Longitudinal Study. *PLoS One*. 2021;16(8). doi:10.1371/journal.pone.0255908
14. Hassaine A, Canoy D, Solares JRA, et al. Learning multimorbidity patterns from electronic health records using Non-negative Matrix Factorisation. *Journal of Biomedical Informatics*. 2020;112:103606. doi:10.1016/j.jbi.2020.103606
15. Hunter ML, Knuiman MW, Musk B, et al. Prevalence and patterns of multimorbidity in Australian baby boomers: the Busselton healthy ageing study. *BMC Public Health*. 2021;21(1). doi:10.1186/s12889-021-11578-y
16. Ibarra-Castillo C, Guisado-Clavero M, Violan-Fors C, Pons-Vigués M, López-Jiménez T, Roso-Llorach A. Survival in relation to multimorbidity patterns in older adults in primary care in Barcelona, Spain (2010–2014): a longitudinal study based on electronic health records. *J Epidemiol Community Health*. 2018;72(3):185-192. doi:10.1136/jech-2017-209984
17. Lenzi J, Avaldi VM, Rucci P, Pieri G, Fantini MP. Burden of multimorbidity in relation to age, gender and immigrant status: a cross-sectional study based on administrative data. *BMJ Open*. 2016;6(12):e012812. doi:10.1136/bmjopen-2016-012812
18. Li J, Green M, Kearns B, et al. Patterns of multimorbidity and their association with health outcomes within Yorkshire, England: baseline results from the Yorkshire Health Study. *BMC Public Health*. 2016;16:649. doi:10.1186/s12889-016-3335-z
19. Linden M, Linden U, Goretzko D, Gensichen J. Prevalence and pattern of acute and chronic multimorbidity across all body systems and age groups in primary health care. *Scientific Reports*. 2022;12(1):272. doi:10.1038/s41598-021-04256-x
20. Mohideen FS, Rajkumar Honest PC, Syed MA, David KV, Abdulmajeed J, Ramireddy N. Prevalence of multimorbidity among adults attending primary health care centres in Qatar: A retrospective cross-sectional study. *Journal of Family Medicine & Primary Care*. 2021;10(5):1823-1828. doi:10.4103/jfmpc.jfmpc_2446_20
21. Mujica-Mota RE, Roberts M, Abel G, et al. Common patterns of morbidity and multi-morbidity and their impact on health-related quality of life: evidence from a national survey. *Quality of Life Research*. 2015;24(4):909-918. doi:10.1007/s11136-014-0820-7
22. Newcomer SR, Steiner JF, Bayliss EA. Identifying Subgroups of Complex Patients With Cluster Analysis. *Am J Manag Care*. 2011;17(8):E324-E332.
23. Nicholson K, Terry AL, Fortin M, Williamson T, Bauer M, Thind A. Prevalence, characteristics, and patterns of patients with multimorbidity in primary care: a retrospective cohort analysis in Canada. *British Journal of General Practice*. 2019;69(686):e647-e656. doi:10.3399/bjgp19X704657
24. Pati S, Swain S, Knottnerus JA, Metsemakers JFM, van den Akker M. Health related quality of life in multimorbidity: a primary-care based study from Odisha, India. *Health & Quality of Life Outcomes*. 2019;17(1):116. doi:10.1186/s12955-019-1180-3
25. Pati S, Swain S, Metsemakers J, Knottnerus JA, van den Akker M. Pattern and severity of multimorbidity among patients attending primary care settings in Odisha, India. *PLoS ONE [Electronic Resource]*. 2017;12(9):e0183966. doi:10.1371/journal.pone.0183966
26. Pefoyo AJK, Bronskill SE, Gruneir A, et al. The increasing burden and complexity of multimorbidity. *BMC Public Health*. 2015;15. doi:10.1186/s12889-015-1733-2

27. Pengpid S, Peltzer K. Multimorbidity in Chronic Conditions: Public Primary Care Patients in Four Greater Mekong Countries. *International Journal of Environmental Research & Public Health [Electronic Resource]*. 2017;14(9):06. doi:10.3390/ijerph14091019
28. Prazeres F, Santiago L. Prevalence of multimorbidity in the adult population attending primary care in Portugal: a cross-sectional study. *BMJ Open*. 2015;5(9):e009287. doi:10.1136/bmjopen-2015-009287
29. Quinones AR, Valenzuela SH, Huguet N, et al. Prevalent Multimorbidity Combinations Among Middle-Aged and Older Adults Seen in Community Health Centers. *J Gen Intern Med*. doi:10.1007/s11606-021-07198-2
30. Saltman DC, Sayer GP, Whicker SD. Co-morbidity in general practice. *Postgraduate Medical Journal*. 2005;81(957):474-480.
31. Shi X, Nikolic G, Van Pottelbergh G, van den Akker M, Vos R, De Moor B. Development of Multimorbidity Over Time: An Analysis of Belgium Primary Care Data Using Markov Chains and Weighted Association Rule Mining. *Journals of Gerontology Series A-Biological Sciences & Medical Sciences*. 2021;76(7):1234-1241. doi:10.1093/gerona/glaa278
32. Singh K, Patel SA, Biswas S, et al. Multimorbidity in South Asian adults: prevalence, risk factors and mortality. *Journal of Public Health*. 2019;41(1):80-89. doi:10.1093/pubmed/fdy017
33. Sinnige J, Korevaar JC, Westert GP, Spreeuwenberg P, Schellevis FG, Braspenning JC. Multimorbidity patterns in a primary care population aged 55 years and over. *Family Practice*. 2015;32(5):505-513. doi:10.1093/fampra/cmv037
34. Soley-Bori M, Bisquera A, Ashworth M, et al. Identifying multimorbidity clusters with the highest primary care use: 15 years of evidence from a multi-ethnic metropolitan population. *British Journal of General Practice*. 2022;72(716):e190-e198. doi:10.3399/BJGP.2021.0325
35. Tan XW, Xie Y, Lew JK, Lee PSS, Lee ES. Patterns of patients with multiple chronic conditions in primary care: A cross-sectional study. *PLoS ONE [Electronic Resource]*. 2020;15(8):e0238353. doi:10.1371/journal.pone.0238353
36. van den Akker M, Buntinx F, Metsemakers JF, Roos S, Knottnerus JA. Multimorbidity in general practice: prevalence, incidence, and determinants of co-occurring chronic and recurrent diseases. *Journal of Clinical Epidemiology*. 1998;51(5):367-375.
37. van Oostrom SH, Picavet HS, van Gelder BM, et al. Multimorbidity and comorbidity in the Dutch population - data from general practices. *BMC Public Health*. 2012;12:715. doi:10.1186/1471-2458-12-715
38. Vargese SS, Mathew E, Johny V, Kurian N, Gayathri AV, Raju AS. Prevalence and pattern of multimorbidity among adults in a primary care rural setting. *Clin Epidemiol Glob Health*. 2020;8(2):482-485. doi:10.1016/j.cegh.2019.10.014
39. Villen N, Guisado-Clavero M, Fernandez-Bertolin S, et al. Multimorbidity patterns, polypharmacy and their association with liver and kidney abnormalities in people over 65 years of age: a longitudinal study. *BMC Geriatrics*. 2020;20(1):206. doi:10.1186/s12877-020-01580-1
40. Violan C, Foguet-Boreu Q, Roso-Llorach A, et al. Burden of multimorbidity, socioeconomic status and use of health services across stages of life in urban areas: a cross-sectional study. *BMC Public Health*. 2014;14:530. doi:10.1186/1471-2458-14-530
41. Vos R, van den Akker M, Boesten J, Robertson C, Metsemakers J. Trajectories of multimorbidity: exploring patterns of multimorbidity in patients with more than ten chronic health problems in life course. *BMC Family Practice*. 2015;16:2. doi:10.1186/s12875-014-0213-6
42. Walker V, Perret-Guillaume C, Kesse-Guyot E, et al. Effect of Multimorbidity on Health-Related Quality of Life in Adults Aged 55 Years or Older: Results from the SU.VI.MAX 2 Cohort. *PLoS One*. 2016;11(12). doi:10.1371/journal.pone.0169282
43. Wong W, Lam CLK, Bian XZ, Zhang ZJ, Ng ST, Tung S. Morbidity pattern of traditional Chinese medicine primary care in the Hong Kong population. *Scientific Reports*. 2017;7(1):7513. doi:10.1038/s41598-017-07538-5
44. Yang X, Zhang J, Chen S, Weissman S, Olatosi B, Li X. Comorbidity patterns among people living with HIV: a hierarchical clustering approach through integrated electronic health records data in South Carolina. *AIDS Care*. 2021;33(5):594-606. doi:10.1080/09540121.2020.1844864
45. Zhang R, Lu Y, Shi L, Zhang S, Chang F. Prevalence and patterns of multimorbidity among the elderly in China: a cross-sectional study using national survey data. *BMJ Open*. 2019;9(8):e024268. doi:10.1136/bmjopen-2018-024268

Supplementary Table 4. Quality assessment of the included studies.

Author, Year	Representativeness	Sample size	Non-respondents	Ascertainment of the exposure	Comparability	Statistical test	Total (maximum 8)
**Bisquera et al., 2021**	1 star	1 star	1 star	2 stars	0 stars	1 star	6 stars
**Diaz et al., 2015**	1 star	1 star	1 star	2 stars	2 stars	1 star	8 stars
**Foguet-Boreu et al., 2015**	1 star	1 star	1 star	2 stars	1 star	1 star	7 stars
**Forslund et al., 2021**	1 star	1 star	1 star	2 stars	0 stars	0 stars	5 stars
**García-Olmos et al., 2012**	1 star	1 star	1 star	2 stars	1 star	1 star	7 stars
**Guisado-Clavero et al., 2018**	1 star	1 star	1 star	2 stars	1 stars	1 star	7 stars
**Machón et al., 2020**	0 stars	0 stars	0 stars	2 stars	2 stars	1 star	5 stars
**Mino-León et al., 2017**	0 stars	1 star	1 star	1 star	0 stars	1 star	4 stars
**Poblador-Plou et al., 2014**	1 star	1 star	1 star	2 stars	1 star	1 star	7 stars
**Prados-Torres et al., 2012**	1 star	1 star	1 star	2 stars	1 star	1 star	7 stars
**Roso-Llorach et al., 2018**	1 star	1 star	1 star	2 stars	1 star	1 star	7 stars
**Stafford et al., 2021**	1 star	1 star	1 star	2 stars	0 stars	1 star	6 stars

**Violán et al., 2018**	1 star	1 star	1 star	2 stars	1 star	1 star	7 stars
**Violán et al., 2019**	1 star	1 star	1 star	2 stars	0 stars	1 star	6 stars
**Violán et al., 2020**	1 star	1 star	1 star	2 stars	0 stars	1 star	6 stars
**Zhu et al., 2020**	1 star	1 star	1 star	2 stars	1 star	1 star	7 stars
